# Application of Multilocus Sequence Typing for the Characterization of *Leptospira* Strains in Malaysia

**DOI:** 10.3390/tropicalmed8020069

**Published:** 2023-01-17

**Authors:** Fairuz Amran, Nurul Atiqah Noor Halim, Ayu Haslin Muhammad, Mohd Khairul Nizam Mohd Khalid, Nur Mukmina Dasiman, Nadia Aqilla Shamsusah, Abdul Khalif Adha Abd Talib, Mohamed Asyraf Noh, Mohammad Ridhuan Mohd Ali, Rohaidah Hashim

**Affiliations:** 1 Bacteriology Unit, Infectious Disease Research Center (IDRC), Institute for Medical Research (IMR), NIH Complex Setia Alam, Shah Alam 40170, Malaysia; 2 Genetic Disorders and Inborn Error of Metabolism (IEM) Unit, Nutrition, Metabolic & Cardiovascular Research Centre (NMCRC), Institute for Medical Research (IMR), NIH Setia Alam, Shah Alam 40170, Malaysia; 3 Department of Earth Sciences and Environment, Universiti Kebangsaan Malaysia, Bangi 43600, Malaysia; 4 Department of Medical Microbiology, Faculty of Medicine and Health Sciences, Universiti Putra Malaysia (UPM), Serdang 43400, Malaysia

**Keywords:** leptospirosis, molecular typing, MLST, allelic profiles, housekeeping genes

## Abstract

Leptospirosis is a common zoonotic disease in tropical and subtropical countries. It is considered an emerging disease in Malaysia and is a notifiable disease. This study was conducted to characterize Malaysian isolates from human, animal and environmental samples via MLST and *rrs2* sequencing in an attempt to develop a Malaysian genotypic database. An existing polymerase chain reaction (PCR)-based MLST scheme was performed to facilitate subsequent sequencing. Out of 46 extracted DNA, 36 had complete MLST profiles whereby all six genes were amplified and sequenced. Most of the pathogenic *Leptospira* genotypes with full MLST profiles were *L. interrogans* serogroup Bataviae (*n* = 17), followed by *L. borgpetersenii* serogroup Javanica (*n* = 9), *L. interrogans* serogroup Sejroe (*n* = 2), *L. interrogans* serogroup Australis (*n* = 2), *L. kirschneri* (*n* = 2), *L. interrogans* serogroup Grippotyphosa (*n* = 1) and *L. interrogans* serogroup Pyrogenes (*n* = 3). Two samples (R3_SER/17 and R4_SER/17) were not closely related with any of the reference strains. For the samples with incomplete MLST profiles, leptospiral speciation was conducted through *rrs2* analysis, in which four samples were identified as *L. borgpetersenii*, five samples were closely related to *L. kmetyi* and one sample was known as *L. yasudae*. This study shows that molecular approaches that combine both MLST and *rrs2* sequencing have great potential in the comprehensive characterization of pathogenic *Leptospira* because they can be performed directly from cultured and clinical samples.

## 1. Introduction

Leptospirosis is a zoonotic infection caused by pathogenic *Leptospira* species and is identified as a global public health concern because of its increased mortality around the world, especially in tropical and humid subtropical regions. This disease can be transmitted to humans through indirect contact with environment contaminated with urine of infected animals, or direct contact with the latter [1]. *Leptospira* can infect a wide range of mammalian hosts, thus leading to a complex and dynamic epidemiology of the disease. Generally, the bacteria are sustained in the renal tubules of long-term carrier hosts, primarily rodents, and are eliminated through their urine. Leptospirosis resembles many other febrile illnesses in its early stages and may include severe diseases with jaundice, pulmonary hemorrhage or central nervous system involvement in its severe form [2,3,4,5].

Previously, the genus *Leptospira* has been classified into three main lineages (pathogenic, intermediate and saprophytic) based on the phylogenetic analyses of 16S *rRNA* genes. However, the lineages have been reclassified as a result of the isolation of more than 30 novel strains recently. This diverse genus is split into four groups: P1 (primary pathogenic strains such as *L. interrogans*, *L. borgpetersenii*, *L. weilii, L. kirschneri, L. santarosai*, *L. mayottensis*, *L. noguchii*, *L. alexanderi*, *L. kmetyi*, *L. astonii*, *L. yasudae*, *L. stimsonii*, *L. astonii*, *L. barantonii*, *L. tipperaryensis, L. adleri, L. ellisii* and *L. gomenensis*), P2 (pathogenic but low-virulence strains such as *L. fainei, L. wolffii*, *L. broomii, L. licerasiae, L. inadai*, *L. hartskeerlii*, *L. dzoumogneensis*, *L. venezuelensis*, *L. selangorensis*, *L. andrefontaineae*, *L. haakeii, L. johnsonii, L. sarikeiensis* and *L. fletcheri*), S1 (primary saprophytes such as *L. biflexa*, *L. vanthielli*, *L. meyeri*, *L. wolbachii*, *L. idonii*, *L. terpstrae*, and *L. yanagawae*) and S2 (new saprophytes such as *L. jelokensis*, *L. kemamanensis*, *L. perdikensis*, *L. congkakensis*, *L. kobayashii* and *L. kanakyensis*) [6,7].

The serovar is a basic identifier characterized based on serological criteria. To date, more than 300 serovars have been described under the *Leptospira* genus [8,9]. Antigenically similar serovars are clustered into >30 different serogroups [10]. Each serovar can infect and adapt to one or more animal hosts [8], making it challenging to track strains whose dynamically infect host and contaminate the environmental niches. Therefore, a complementary leptospiral typing is essential for identifying potential clusters, transmission pathways and host reservoir.

However, serological data are frequently presented without genetic data, or vice versa, which reduces the effectiveness of public health surveillance and investigations [11]. Molecular techniques are more advantageous compared to serological testing in clinical diagnostics due to their speed and higher sensitivity in the early stages of infection [12]. The two commonly applied molecular techniques are pulsed-field gel electrophoresis (PFGE) and multilocus sequence typing (MLST). MLST is robust and reproducible and can even be applied directly in clinical samples to type the infecting *Leptospira* [13,14,15]. MLST is widely used to characterize the subspeciation of *Leptospira* [16]. This method is a simple polymerase chain reaction (PCR)-based technique that identifies strains from a sequence of six or seven housekeeping with extremely slow evolutionary change [16,17]. For each strain of a specific species, the genes are amplified and sequenced. Each sequenced gene is then compared to every previously uploaded sequence (alleles) at that locus. Then, each of the six or seven loci is given an allele number. The allelic profile is interpreted using a combination of the six or seven allele numbers (assigned as sequence type, ST), which is used to describe the strain [18,19]. In this study, we aimed to characterize Malaysian isolates from human, animal and environmental samples via MLST and *rrs2* sequencing in an attempt to develop a Malaysian genotypic database.

## 2. Materials and Methods

### 2.1. Sample Collection

Blood samples from patients suspected of leptospirosis infection were collected from local hospitals (*n* = 13), animal blood and environmental samples were obtained from rodents caught in wet markets (*n* = 30), wild dogs (*n* = 2) and soil (*n* = 1), respectively.

### 2.2. Isolation of Leptospira sp. and Dark-Field Microscopy Observation

To culture leptospires from rodent samples, kidneys from captured rodents were macerated in 2 mL of semi-solid Ellinghausen and McCullough modified by Johnson and Harris (EMJH) media and were allowed to settle for 20 min. The mixtures were then filtered into 5 mL of new media using 0.45 μm and 0.22 µm sterile syringe filters. For blood and urine samples collected from patients and animals, 2–3 drops of each sample were directly inoculated into 5 mL of EMJH media and incubated at 30 °C. Cultures were examined under a dark-field microscope at intervals of seven days. Leptospira were identified by their morphology and motility. Leptospira are very thin, long, and have hooked ends. Leptospires rotate on their longitudinal axis rapidly, moving forward and backward. The cultures were considered *Leptospira*-negative if there was no growth of the bacteria after three months and could thus be discarded.

### 2.3. DNA Extraction

For the MLST performed directly from clinical samples, DNA was extracted from 400 μL whole blood samples using the Maxwell 16 Automated Research Instrument. DNA from Leptospira isolates was extracted using the DNeasy Blood and Tissue Kit (Qiagen, Valencia, CA, Spain) following the manufacturer’s recommendation. Positive *Leptospira* cultures were extracted using the boiling method in which 2 mL of each sample was pelleted at 13,000× *g* for 30 min at 4 °C, washed two times with 1000 μL of phosphate-buffered saline (PBS), and re-suspended in 150 mL of TE buffer. Eluates were boiled at 100 °C for 7 min. DNA quantification was performed using a Thermo Scientific NanoDrop™ spectrophotometer to measure the purity and concentration of DNA. All DNA samples were stored in −20 °C to prevent degradation [20].

### 2.4. Detection and Characterization of Leptospira sp. in Cultured and Non-Cultured Samples Using rrs2 Genes

The following forward and reverse primers of the *rrs2* gene were used: 5′-CATGCAAGTCAAGCGGAGTA-3′ and 5′-GCATCGAGAGGAATTAACATCA-3′ [21]. The PCR reactions were prepared containing 1 × PCR buffer, 0.2 mM of dNTPs, 0.5 μm of each primer, 1.5 mM of MgCl2, 0.5 U of Taq DNA polymerase (Qiagen, Hilden, Germany), DNA template and water (adjusted to 25 μL final reaction volume). The cycling conditions consisted of initial denaturation at 94 °C (3 min), followed by 35 cycles of 94 °C (1 min), 54 °C (1 min), 72 °C (2 min) and a final extension at 72 °C (10 min). The PCR amplicons were analyzed by electrophoresis on 1% agarose gel, purified and sequenced in forward direction via the Sanger sequencing method. The sequences were trimmed and compared to the GenBank database using the online BLAST webtool available at http://www.ncbi.nih.gov (accessed on 7 August 2017).

### 2.5. Detection and Characterization of Leptospira sp. in Cultured and Non-Cultured Samples through the Mlst

A previously published MLST scheme was adapted, based on the amplification of six genes, namely *adk*, *icdA*, *LipL32*, *LipL41*, *rrs2* and *secY* (Table 1) [22]. The protocol was prepared using HotStar Taq Master Mix in a 25 μL reaction, consisting of a 100 nmol MgCl2 for *tpiA* only, 5 pmol of primers, and DNA template (40–60 ng of DNA from isolates or 5 μL of DNA from clinical samples). Cycling conditions were similar to the published guidelines, except for an additional 15-min initial 95 °C incubation to activate the enzyme. Restriction endonuclease-PCR was conducted if no bands were produced during the first trial to facilitate subsequent sequencing.

The PCR amplicons were analyzed using 1% agarose gel electrophoresis and sequenced. Individual sequences were assembled using Genius Pro 5.4.6 software. The assembled sequences were trimmed and aligned with the reference sequences downloaded from the sequences available in the database. The phylogenetic analysis of the neighbor-joining phylogenetic trees was constructed from the concatenated (2980 bp) sequences in the order of *adk*, *icdA*, *LipL32*, *LipL41*, *rrs2* and *secY* using Molecular Evolutionary Genetic Analysis software version 7.

## 3. Results

**Figure 1:**
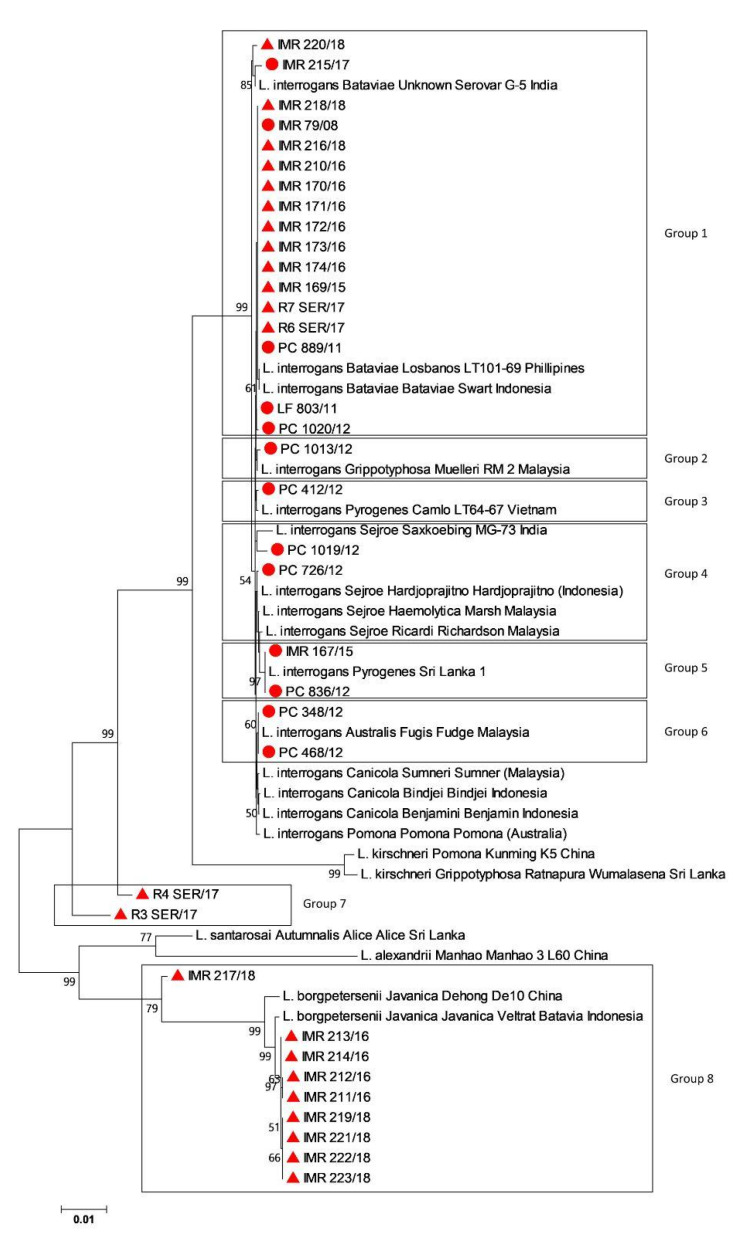
Genetic relatedness comparing isolates from animal (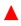) and human (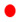) samples with reference strains from different countries based on the concatenated sequences of the six housekeeping genes using neighbor-joining phylogenetic trees with bootstraps of 100.

Out of 46 extracted DNA, 36 had complete MLST profiles, of which all six genes were amplified and sequenced. Most of the pathogenic *Leptospira* genotypes with full MLST profiles were *L. interrogans* serogroup Bataviae (*n* = 17), followed by *L. borgpetersenii* serogroup Javanica (*n* = 9), *L. interrogans* serogroup Sejroe (*n* = 2), *L. interrogans* serogroup Australis (*n* = 2), *L. kirschneri* (*n* = 2), *L. interrogans* serogroup Grippotyphosa (*n* = 1) and *L. interrogans* serogroup Pyrogenes (*n* = 3). Two samples (R3_SER/17 and R4_SER/17) were not closely related with any of the reference strains (Figure 1). Table 2 shows the estimates of the average evolutionary divergence over sequence pairs within the groups.

**Figure 2:**
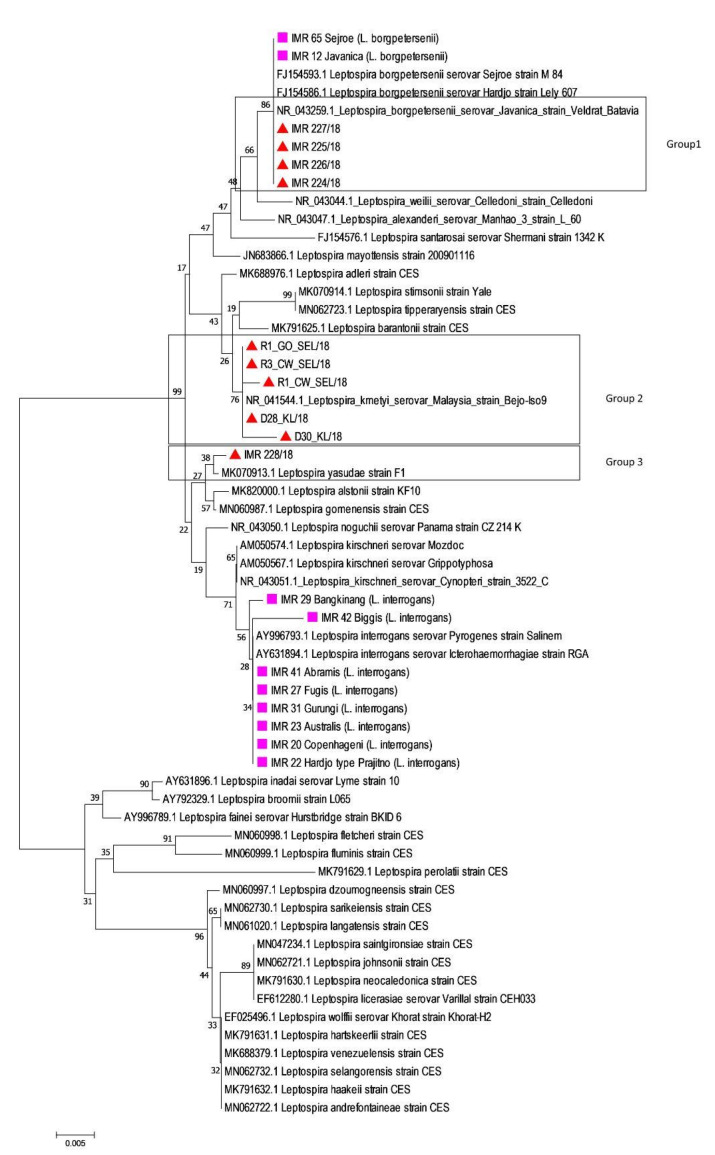
Genetic relatedness comparing strains from rodents, dogs and soil samples (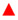) to old reference Malaysian *Leptospira* isolates (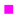) and NCBI reference strains based on *rrs2* genes using neighbor-joining phylogenetic trees with bootstraps of 100.

An *rrs2* phylogenetic tree was constructed for all 10 sample strains with incomplete MLST profiles (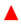) (Figure 2). The *rrs2* analysis showed a clustering of *Leptospira* into distinct species for both culture-positive and culture-negative samples. Four samples were identified as *L. borgpetersenii*, five samples were closely related to *L. kmetyi* and one sample was known as *L. yasudae*. Table 3 shows the estimates of the average evolutionary divergence over sequence pairs within the groups.

## 4. Discussion

The identification and typing of the infecting leptospiral can hint the animal source of the infections, as certain serovars are associated with either a single or an exclusive group of mammalian species [4,23]. Such information provides a vital contribution to the planning and implementation of effective prevention strategies [4,23,24]. As 16S *rRNA* gene sequencing has poor discriminatory strength and whole genome sequencing is currently impractical for routine strain typing, additional gene targets, such as *secY* and *glmU* genes, have been utilized to type *Leptospira* species [6,11,25,26]. *LipL32* gene PCR sequencing has also been used extensively because it is a robust target for leptospirosis diagnosis and only detects pathogenic leptospires [27]. However, this technique lacks selective strength due to the conserved nature of the *lipL32* gene sequence [11]. Furthermore, due to sequence polymorphism, the *lipL32* gene PCR cannot detect P2 leptospires [27]. Thus, three different 6- or 7-loci MLST schemes have been most frequently utilized in typing *Leptospira* because of its ability to differentiate between seven pathogenic species of *Leptospira* up to the serovar level, in addition to the potential phylogenetic evidence that provide information on the relatedness between leptospiral species [16,21,27,28]. Ideally, *Leptospira* typing is carried out using isolates. Albeit culture is rarely performed in routine microbiology laboratories due to its low sensitivity and long incubation time, it plays a key role in elucidating both local and global epidemiology of infection [28,29,30,31].

As shown in Figure 1, most of the rodent samples from Kuala Lumpur wet markets and cities were closely related to *L. interrogans* serogroup Bataviae (Group 1) and *L. borgpetersenii* serogroup Javanica (Group 8), with a mean genetic distance within the groups of 0.0028 and 0.0049, respectively (Table 2). Comparable results were obtained by Benacer et al. [32] and Blasdell et al. [33]: *L. borgpetersenii* and *L. interrogans* were highly prevalent among urban rodents in Kuala Lumpur and Sarawak, respectively, indicating the potential role of the rat population as the maintenance hosts in the local urban transmission of *Leptospira*. Further, wet markets are a prime location for the spread of many infectious diseases and numerous studies have recently associated leptospirosis with them [34,35,36]. Leptospirosis may spread to humans due to a number of circumstances, including poor hygiene and management. Moreover, like other parts of the world, rapid urbanization, and high density of population in urban areas have attracted primary hosts of this disease, allowing them to harbor *Leptospira*, which then act as a persistent reservoir of infection to both livestock and humans cohabiting with rats [33]. Thus, regular monitoring of infections in wild rodents is essential to address important health problems and provide information that can lead to an effective public health policy [37].

Based on our results, *L. interrogans* was shown to be the main infecting *Leptospira* species in humans, with a broad range of infecting serogroups (Figure 1). Among these commonly identified serogroups in Malaysia, the most predominant serovars in humans were Bataviae and Sejroe (Figure 1). Interestingly, in the northeastern state of Malaysia, Shafei et al. [38] reported two predominant serogroups, Javanica and Bataviae, among town service laborers. Conversely, Rafizah et al. [39] reported the serogroup Sejroe as the common serogroup detected in febrile inpatient cases. Additionally, two samples of group 7 (R3_SER/17 and R4_SER/17) were not closely related with any of the reference strains based on the alignment of concatenated sequences of MLST Scheme 3 (Figure 1). This is because information on reference strains in the MLST database is limited to *L. interrogans* and *L. kirschneri* [16] and no allelic profiles have been assigned to new leptospiral strains. Furthermore, concatenated sequences of R3_SER/17 and R4_SER/17 might be unique with allelic variation at each locus, hence unable to find the exact/nearest match when compared with the database.

In this study, we were unable to isolate *Leptospira* from certain human and animal samples, although they were PCR-positive. We speculated that these subjects could become infected with isolates that favored natural conditions compared to the standard culture medium used in the laboratory, as *Leptospira* are fastidious microorganisms with high growth requirements. Not only that, clinical specimens often have very low bacterial burden [11]. Boonsilp et al. [40] also pointed out some possible explanations for patients who were PCR-positive but culture-negative. There is a possibility that the patients might have received an empirical antimicrobial drug. Furthermore, due to fluctuations in ambient temperature and delay, *Leptospira* might also become non-viable in the blood tube during the storage or transportation to the laboratory.

Therefore, in such instances, a direct identification of infecting *Leptospira* from clinical samples is favored due to the clinical and epidemiological importance [27,41,42]. However, because a very low amount of DNA is present in clinical samples, obtaining complete MLST results is laborious. For example, Chiani et al. [43] applied a seven-locus scheme to clinical samples, serum and whole blood from leptospirosis patients but failed to obtain complete MLST profiles. Thus, Boonsilp et al. [40] proposed a nested PCR for the *rrs* genes to detect and speciate pathogenic *Leptospira* in blood from patients with culture-negative leptospirosis. Thus, in our study, as *rrs2* gene sequence analysis could be a reliable screening for species identification in culture-negative samples, isolates with incomplete MLST profiles were also included.

A phylogenetic tree based on *rrs2* sequences (Figure 2) showed that *Leptospira* in all rodent samples were closely related to *L. borgpetersenii*, similar to the result in Group 8 (Figure 1). The isolates obtained from three cattle, two dogs, and one environmental sample were closely related to *L. kmetyi* serovar Malaysia (Group 2) and *L. yasudae* (Group 3) (Figure 2), with a mean genetic distance within group of 0.001 and 0.002, respectively (Table 3). These animals could become infected with *Leptospira* because of constant exposure to contaminated soil, as *L. kmetyi* was mostly isolated from environmental samples [44,45,46]. In addition, a novel species, *L. yasudae* was also isolated from the soil of an ex-situ wild animal conservation area in Malaysia recently [47]. Although both species are uncommonly isolated from clinical samples, *L. kmetyi* and *L. yasudae* have been classified under the P1 group based on the phylogenetic analysis of 16S *rRNA* gene as well as their genome compositions [7,44,47,48].

In this study, the inability of our local isolates to obtain complete information from all six MLST loci might be due to insufficient quantity of bacterial DNA in clinical specimens, which is necessary for MLST amplification. This is because *Leptospira* are only present in the blood within the first few days (4–6 days) post infection while in the urine, the bacteria can be detected after one week of illness [49,50]. Thus, the type of specimen and sampling window after symptom onset may prove crucial for MLST diagnosis directly from specimens. Furthermore, incomplete MLST profiles of our local isolates is attributable to the fact that they might be genetically unique, as the designing of primers was based on sequences from *Leptospira* recovered from other parts of the world. Conversely, the ability to recover the *rrs2* sequence despite the incomplete coverage of six MLST loci could be due to the presence of multiple copies of the 16S *rRNA* gene in the leptospiral genome compared to the single copy genes used for MLST. Although a partial *rrs2* sequence is adequate for molecular speciation, further sub-classification would require sequence information from complete MLST profiles.

## 5. Conclusions

This study indicates that molecular approaches by both MLST and *rrs2* sequencing have immense potential in the comprehensive characterization of pathogenic *Leptospira* because they can be performed directly from clinical samples. MLST is also useful, especially in molecular epidemiological studies, for tracking the evolution and spread of this important human pathogen.

## Figures and Tables

**Table 1:** Oligonucleotide sequence for both forward and reverse primers in MLST Scheme 3.

Primers	Oligonucleotide Sequence (5′–3′)
*adk*	F-GGG CTG GAA AAG GTA CAC AA
	R-ACG CAA GCT CCT TTT GAA TC
*icdA*	F-GGG ACG AGA TGA CCA GGA T
	R-TTT TTT GAG ATC CGC AGC TTT
*lipL32*	F-ATC TCC GTT GCA CTC TTT GC
	R-ACC ATC ATC ATC ATC GTC CA
*lipL41*	F-TAG GAA ATT GCG CAG CTA CA
	R-GCA TCG AGA GGA ATT AAC ATC A
*rrs2*	F-CAT GCA AGT CAA GCG GAG TA
	R-AGT TGA GCC CGC AGT TTT C
*secY*	F-ATG CCG ATC ATT TTT GCT TC
	R-CCG TCC CTT AAT TTT AGA CTT CTT C

**Table 2:** Estimates of the average evolutionary divergence over sequence pairs within groups based on MLST analysis.

Group No.	Mean Distance (within Groups)	Standard Error (S.E)
1	0.0028	0.0004
2	0.0007	0.0005
3	0.0007	0.0005
4	0.0017	0.0008
5	0.0000	0.0000
6	0.0000	0.0000
7	0.0181	0.0024
8	0.0049	0.0011

**Table 3:** Estimates of the average evolutionary divergence over sequence pairs within groups on *rrs* genes.

Group No.	Mean Distance (within Groups)	Standard Error (S.E)
1	0.000	0.000
2	0.001	0.001
3	0.002	0.002

## Data Availability

Available upon request.
